# An Evaluation of a Modified CTAS at an Accident and Emergency Department in a Developing Country

**DOI:** 10.1155/2018/6821323

**Published:** 2018-05-02

**Authors:** Shalini Pooransingh, L. K. Teja Boppana, Isaac Dialsingh

**Affiliations:** ^1^Faculty of Medical Sciences, The University of the West Indies, St. Augustine Campus, Champs Fleurs, Trinidad and Tobago; ^2^Faculty of Science and Technology, The University of the West Indies, St. Augustine Campus, St. Augustine, Trinidad and Tobago

## Abstract

**Objectives:**

To review the modified Canadian Triage and Acuity Scale used in an accident and emergency department in Trinidad and Tobago.

**Design and Methods:**

A cross-sectional study was carried out. Times from assignment of triage category to being seen by a physician were collected from the patient notes on the days of presentation and compared to the reference standards. Times from decision to admit to obtaining a bed were also recorded.

**Results:**

200 patients were included in the study. The median waiting time for patients in the immediate/blue category was 3 minutes (range = 3); for the red category, it was 31.2 minutes (range = 121.8); in the yellow category, it was 61.8 minutes (range = 805.2). The overall admission rate was 30.5%, with an admission rate of 25% for the blue category; 20% of patients in the red category waited more than 4 hours for a hospital bed.

**Conclusion:**

The patients assigned to the blue category were being seen almost immediately. Less critical persons wait longer than the reference times and this may be due to structural factors such as staffing. The admission rates per category highlighted a low admission rate for the blue category (25%), which is unusual. This study highlights the need for a further study to review clinical presentation, assignment to triage category, and outcomes.

## 1. Introduction

“Triage” derived from the French word for “sort” is a process by which patients are prioritized and classified according to the type and urgency of the condition [1]. Accident and emergency (A&E) Departments are the first port of call for many patients. For example, the A&E Department at Port of Spain General Hospital (POSGH), where this study was carried out, provides care and treatment to patients with a wide variety of illnesses [2], ranging from motor vehicle accidents and gunshot wounds to presentations such as back pain. Applying triage can lead to safe and efficient utilization of an emergency department [3]. On presentation to the A&E Department, a triage doctor/nurse follows a triage system to determine the urgency by which a patient needs to be seen by healthcare professionals and to designate appropriate resources to care for the identified problem [4]. Triage attempts to have the most critically ill patients seen first with an overall reduction in waiting time [5]. There are numerous triage instruments in use around the world, namely, the Australasian Triage Scale, the Canadian Triage and Acuity Scale, the Manchester Triage System, and the Emergency Severity Index. Triage instruments with 5 levels are superior to those with 3 levels in both validity and reliability [6].

The Canadian Triage and Acuity Scale (CTAS) [7] is a 5-level triage system (level I = resuscitation (blue, to be seen immediately), level II = emergent (red, to be seen <15 min), level III = urgent (yellow, to be seen <30 min), level IV = less urgent (green, to be seen <60 min), and level V = nonurgent (white, to be seen <120 min)). The CTAS is based on a comprehensive list of patients' complaints used to ascertain the triage level. Each complaint has been described in detail covering high-risk indicators [8].

At the POSGH, the CTAS was adapted for use with one major change. Levels III and IV patients were grouped together and triaged under yellow, and level V patients were triaged under green. This resulted in a 4-level system with patients triaged under yellow (levels III and IV) having to be seen within 60 minutes and those triaged under green (level V) having to be seen within 120 minutes. There was no change to levels I and II categories. The person responsible for triaging patients is a medical doctor. The aim of this paper was to evaluate the adapted CTAS system in operation at the Port of Spain General Hospital against the reference standards.

## 2. Methods

A cross-sectional study was carried out. The study was conducted during the three-month period from April to June 2013. The study population comprised all patients who presented to the A&E Department of the Port of Spain General Hospital during the study period. The study sample included 200 patients who were selected from each shift on all days of the week (i.e., from Monday to Sunday between the hours of 8:00 am to 4:00 pm, 4:00 pm to 12:00 am, and 12:00 am to 8:00 am).

The department handles approximately 200 patients per day. In terms of treatment spaces, there are two resuscitation spaces, six critical bay spaces, four patient examination rooms, an asthma room, and an observation room with twelve spaces for patients waiting to be discharged, waiting for results, and waiting for possible same day discharge or admission to a ward.

In terms of staffing, in the morning shift from 8 am to 4 pm, there are two registrars and ten house officers on site; during the 4 pm to midnight shift, there are two registrars and nine house officers on site and from midnight to 8 am there are four house officers on site with the registrar and consultant accessible by telephone initially.

There are two main areas where patients are located: patients assigned to the resuscitation/emergent (blue and red) categories were located in one part of the department and urgent, less urgent, and nonurgent (yellow and green) patients were located in another part of the department. Every fifth patient was chosen from each of the two groups and included in the study. Persons who were suffering from diminished mental capacity were excluded.

Data collection included information on triage times, which was taken from the patient notes. In addition, data on the time a person had to wait for a bed once a decision to admit was made were also taken from the patient records. Data were entered into a Microsoft Excel spreadsheet and subsequently analysed using Statistical Package for the Social Sciences (SPSS) version 23. A significant result was indicated by a *p* value < 0.05.

Ethical approval was obtained from the University of the West Indies, St. Augustine, and from the North West Regional Health Authority, the administrative health authority for the Port of Spain General Hospital.

## 3. Results

A total of 200 patients were included in the study.

### 3.1. Patient Characteristics

There was an approximate male to female ratio of 1 : 1 with the age group of 30–45 years predominating. The majority of patients (71.5%) were of African-Trinidadian origin. There was a significant difference in the proportions of patients in each triage category (Chi-square = 176.375; df = 3; *p* value < 0.001). We investigated whether there were any significant associations between triage category and gender, age, and ethnicity. Fisher's exact test was used to determine whether there were significant differences.  Table 1 shows that there were no significant associations between these variables and triage category, since the *p* values were all greater than the 0.05 level of significance for this study.

### 3.2. Waiting Times per Triage Category

The waiting times for each triage category were reviewed. The data in Table 1 shows that those assigned to the blue category had the least waiting time, a median of 0.05 hours with a range of 0.05; the reference would be that patients were seen immediately. The next serious triage category, red, saw a median waiting time of 0.52 hours and a range of 2.03. Patients in the yellow category had the longest waiting time with a median of 1.03 hours (range = 13.42).

A Kruskal-Wallis test was conducted to evaluate differences among the median waiting times of the four triage categories. The test, which was corrected for tied ranks, was significant (Kruskal-Wallis *H* = 15.386, *p* value = 0.002).

Mann–Whitney's pairwise tests were done to determine any significantly different pairwise differences among the four groups. We controlled for type I error across tests using Holm's sequential Bonferroni approach. The results of these analyses indicated that there were significant differences in the median waiting times among patients in the blue and red, blue and yellow, and blue and green categories. The median waiting times for all the other pairs of categories were not statistically significant.

### 3.3. Proportion of Patients Admitted in Each Assigned Triage Category

To obtain an indication of the validity of the initial assignment to a triage category, as a proxy, we reviewed the proportion of patients who were admitted per triage category for which the data were in the notes. In total, 63 patients were admitted, but the relevant data were contained in 58 records.  Table 2 shows the findings.

### 3.4. Waiting Times for Patients Admitted to the Ward

To determine the sense of urgency applied in keeping with the assigned triage category, we calculated the time spent waiting for a bed from when the doctor made a decision to admit the patient. The findings are shown in Table 3.

## 4. Discussion 

This study was carried out in an A&E Department in a capital city during a three-month period. Trinidad and Tobago, a small developing country, enjoys a tropical climate with two seasons, the dry season from January to June and a rainy season from July to December. There is little temperature variation throughout the year. There is therefore little, if any, variation in presentations to the A&E Departments throughout the year with presentations ranging from motor vehicle accidents and gunshot wounds to back pain [2].

The analysis revealed that the predominant ethnic group presenting was the African-Trinidadian group. This is representative of the city where the A&E Department is located. There were no differences observed in patient characteristics across all triage categories, which can be considered a positive finding in terms of equity of access.

When comparing the waiting times per triage category against the reference, the study findings reveal (Table 1) that the patients assigned to the blue (immediate) category are being seen almost immediately; patients categorised in this stratum are being seen within 3 minutes on average with a range of 3 minutes. However, patients assigned to the red category who should be seen in less than 15 minutes report a median waiting time of 31 minutes with a range of 121.8. It appears that some patients are not being seen according to the standard. The patients assigned to the yellow category at POSGH need to be seen within 60 minutes. Their median waiting time was 61.8 minutes with a range in excess of 800. The patients placed in the green category with a standard of needing to be seen within 120 minutes are also not all being seen according to the standard, although their median waiting time was 37.2 minutes.

Reasons for the nonadherence to the triage waiting times standards could be health system factors such as staff shortages due to shortage of funds resulting in no hiring of staff, staff absenteeism during shifts, or staff unfamiliarity with the triage system, since there are no formal systems in place in Trinidad and Tobago compared to the United Kingdom, for example, where hospital performance is routinely monitored. Indeed, the majority of doctors on site during shifts are junior hospital doctors with no registrars on site from midnight. It was observed that many patients assigned to the yellow category presented during shifts when there were less doctors on duty which could have resulted in the delays [personal communication]. It has been recognized that the volume of patients presenting to the A&E Departments cannot be planned and hence sometimes the resources may be overwhelmed.

Informing patients about the triage system could help reduce complaints on their part with regard to waiting times but also can give a sense of autonomy over their experience at the A&E Department. Additionally, if healthcare staff know that the patient is aware of their triage category and the expected waiting time, efficiency may improve all around.

In terms of the proportion of patients admitted to hospital by triage category, it was curious that only 25% of patients were admitted in the blue category. One would expect patients who fall into this triage category to have illnesses of such severity that they would require hospital admission. Our study did not collect data to confirm that this was this case. However, the A&E Department has provision for stabilizing patients, for example, patients suffering from acute severe asthma who may be discharged after stabilisation, and perhaps this may have contributed to the lower than expected admission rate in the blue category.

Furthermore, in the analysis of how long patients waited for a bed once a decision to admit was made (Table 3), of ten patients in the red category, eight patients were waiting more than 1 hour with two waiting more than 4 hours. This does not correlate with a serious triage category unless the patient was stabilized while in A&E Department which this study did not review. The United Kingdom's National Health Service states that a minimum of 95% of patients attending an A&E Department should be admitted, transferred, or discharged within 4 hours of their arrival [9]. In our study, 24% waited more than 4 hours for a bed. We did not record times for discharge or transfers.

Although this study shows adherence to standards for the immediate triage category, we used two parameters as proxies to attempt to validate the initial assignment to a triage category. As previously discussed, the proportion of patients admitted per category and the waiting times in A&E Department for a bed suggest that a more in-depth approach with more information is needed. In a future study, we need to note the presumptive diagnosis and vital signs at triage assignment. This is important as there has been disagreement among experts on determining urgency even when the same criteria are used [10]. A retrospective review looking at triage category and clinical outcome could also be used to assess the triage system and this is another approach that we may adopt in future reviews of the system. The Manchester Triage System was evaluated and has been found to be a sensitive tool for those requiring critical care who are ill on arrival at A&E Department [11]. The five-level CTAS system has been documented to be outstanding in terms of its validity and reliability [12].

### 4.1. Limitations of the Study

We sampled 200 patients from the A&E Department from all days of the week and from all shifts. We note that this sample is relatively small in statistical terms; however, our findings have shown that certain aspects of the system seem to be working, but the waiting times for a bed appear longer than we would expect patients to have to wait.

## 5. Conclusion

In conclusion, the persons assigned to blue category are falling within 3 minutes of the standard. However, less critical patients need to wait, which may be a result of staffing complement. Although the POSGH employed a modified 4-level CTAS when the study was carried out, the 5-level triage systems are the gold standard [12]. In keeping with clinical governance, the study findings should be given serious consideration. An admission rate of 25% for the blue category suggests a need to review the triage assignment and to review the outcomes of the patients. While reviewing the triage assignment, interrater agreement would be a useful measure. An overall 30.5% admission rate seems to be high compared to other settings [13, 14]. But what about the other 69.5%? The majority would have been discharged and/or given a clinic follow-up appointment. There is always the question of whether some patients should be attending primary care services rather than the hospital's A&E Department. The arguments against this would include limited access to primary care services after working hours for persons who fall ill after 4 pm and for those who are employed during the day, and some conditions such as sprains and fractures are best seen at the A&E Department. Access to blood tests and imaging is also not readily available in all primary care settings; thus patients prefer to attend the A&E Department as a “one stop shop” where all diagnostic facilities and treatments are available. A study documenting clinical signs and symptoms at presentation, the assigned triage category in addition to patient outcome, and reasons for long A&E Department stays would be a next step to evaluate the system.

## Figures and Tables

**Table 1:** Patients' factors (age, sex, and ethnicity) and waiting times according to triage category.

	Triage				*p* value
	Blue	Red	Yellow	Green	
Gender					
Male	2 (2.2%)	12 (13.0%)	52 (56.5%)	26 (28.3%)	0.142^*∗*^
Female	2 (2.2%)	9 (9.0%)	72 (72.0%)	17 (17.0%)	

Age (years)					
17–29	2 (4.5%)	6 (13.6%)	29 (65.9%)	7 (15.9%)	0.315^*∗*^
30–45	1 (1.8%)	6 (10.7%)	33 (58.9%)	16 (28.9%)	
46–60	0 (0.0%)	2 (3.9%)	37 (72.5%)	12 (23.5%)	
>60	1 (2.5%)	7 (17.5%)	25 (62.5%)	7 (17.5%)	

Ethnicity					
African-Trinidadians	4 (2.9%)	10 (7.4%)	91 (66.9%)	31 (22.8%)	0.204^*∗*^
Indian-Trinidadians	0 (0.0%)	7 (20.6%)	21 (61.8%)	6 (17.6%)	
Other	0 (0.0%)	4 (19.0%)	11 (52.4%)	6 (17.6%)	

Waiting time (hours)					
Median	0.05	0.52	1.03	0.62	0.002^*∗∗*^
Range	(0.05)	(2.03)	(13.42)	(10.27)	

^*∗*^ *p* values are based on Fisher's exact test. ^*∗∗*^*p*-value based on the one-way nonparametric test for independent samples (Kruskal-Wallis test).

**Table 2:** The number and proportion of patients who were admitted to hospital in each triage category.

Triage category	Total number of patients	Number of patients admitted	Proportion admitted by triage category	Proportional representation by category
Blue	4	1	25%	1.7%
Red	21	10	48%	17%
Yellow	124	40	32%	69%
Green	43	7	16%	12%

Total	192	58	—	—

**Table 3:** Triage category of patients admitted and the time (hours) they spent waiting for a bed on the ward.

Triage category	<1 h	>1, <2 h	>2 h, <3 h	>3 h, <4 h	>4 h	Total
Blue	1	—	—	—	—	1
Red	2	3	2	1	2	10
Yellow	8	10	7	4	11	40
Green	2	2	1	1	1	7
